# High Prevalence of HIV-1 CRF01_AE Viruses among Female Commercial Sex Workers Residing in Surabaya, Indonesia

**DOI:** 10.1371/journal.pone.0082645

**Published:** 2013-12-18

**Authors:** Tomohiro Kotaki, Siti Qamariyah Khairunisa, Septhia Dwi Sukartiningrum, M. Vitanata Arfijanto, Takako Utsumi, Irine Normalina, Retno Handajani, Prihartini Widiyanti, Musofa Rusli, Retno Pudji Rahayu, Maria Inge Lusida, Yoshitake Hayashi, Nasronudin, Masanori Kameoka

**Affiliations:** 1 Indonesia-Japan Collaborative Research Center for Emerging and Re-emerging Infectious Diseases, Institute of Tropical Disease, Airlangga University, Surabaya, Indonesia; 2 Center for Infectious Diseases, Kobe University Graduate School of Medicine, Hyogo, Japan; 3 Faculty of Medicine, Airlangga University, Surabaya, Indonesia; 4 Department of International Health, Kobe University Graduate School of Health Sciences, Hyogo, Japan; National Institute for Viral Disease Control and Prevention, CDC, China

## Abstract

**Background:**

Human immunodeficiency virus (HIV) infection and acquired immune deficiency syndrome (AIDS) cause serious health problems and have an impact on the Indonesian economy. In addition, the rapid epidemic growth of HIV is continuing in Indonesia. Commercial sex plays a significant role in the spread of HIV; therefore, in order to reveal the current HIV prevalence rate among commercial sex workers (CSWs), we conducted an epidemiological study on HIV infection among CSWs residing in Surabaya, the capital of East Java province of Indonesia with large communities of CSWs.

**Methodology/Principal Findings:**

The prevalence of HIV infection among 200 CSWs was studied. In addition, the subtype of HIV type 1 (HIV-1) and the prevalence of other blood-borne viruses, hepatitis B virus (HBV), hepatitis C virus (HCV) and GB virus C (GBV-C), were studied. The prevalence rates of HIV, hepatitis B core antibody, hepatitis B surface antigen, anti-HCV antibodies and anti-GBV-C antibodies were 11%, 64%, 4%, 0.5% and 0% among CSWs involved in this study, respectively. HIV-1 CRF01_AE viral gene fragments were detected in most HIV-positive samples. In addition, most CSWs showed low awareness of sexually transmitted diseases and had unprotected sex with their clients.

**Conclusions/Significance:**

The HIV prevalence rate among CSWs was significantly higher than that among the general population in Indonesia (0.2–0.4%). In addition, CSWs were at a high risk of exposure to HBV, although chronic HBV infection was less frequently established. Our results suggest the necessity of efficient prevention programs for HIV and other blood-borne viral infections among CSWs in Surabaya, Indonesia.

## Introduction

According to the report from the Joint United Nations Programme on HIV/AIDS (UNAIDS), the number of people newly infected with human immunodeficiency virus (HIV) continues to fall year by year globally; however, rapid epidemic growth of HIV is continuing in several countries [1]. Among Southeast Asian countries, the annual incident rate of HIV infection has declined in many countries, including Cambodia, Malaysia, Myanmar, Nepal and Thailand, whereas it has continuously increased in countries such as Indonesia, Bangladesh and the Philippines [1], [2]. In Indonesia, the number of people living with HIV was estimated to be 380,000 at the end of 2011, and 55,000 people were newly infected with HIV in 2011. In addition, the estimated number of people living with HIV markedly increased (3166.7%) from 12,000 people in 2001 to 380,000 people in 2011 in this country [1]. Furthermore, uncertainty in the estimation of the number of people living with HIV in Indonesia is apparent [1], suggesting the importance of accumulating more epidemiological data in Indonesia.

HIV is a blood-borne virus that spreads through contaminated blood and other body fluid. In Indonesia, the sharing of needles and syringes is considered to be a major high-risk practice for HIV transmission among injecting drug users (IDUs) [3], [4]. In addition, the sexual transmission of HIV has also apparently increased in Indonesia [5], [6]. Commercial sex plays a significant role in the spread of HIV [7]; however, the coverage of HIV prevention programs among commercial sex workers (CSWs) is reported to be less than 25% in Indonesia [1]. In this report, we conducted an epidemiological study on the prevalence of HIV among CSWs residing in Surabaya, the capital of East Java province of Indonesia with large communities of CSWs. In addition, viral subtyping to reveal the prevalent strains of HIV-1 as well as an epidemiological study on the prevalence of other blood-borne viruses, hepatitis B virus (HBV), hepatitis C virus (HCV) and GB virus C (GBV-C), were carried out.

## Methods

### Ethics statement

This study was conducted with approval from the institutional ethics committees of the Institute of Tropical Disease and the Institute for Research and Public Service, Airlangga University and Kobe University Graduate School of Medicine as well as with written informed consent from study participants.

### Study participants and sample collection

Two hundred CSWs with an age range of 18–51 years old (median 32 years old), consisting of 13, 124 and 63 individuals residing in two urban areas, urban area 1 and 2, and a rural area of Surabaya, respectively, were enrolled in this study (Table 1). We had randomly recruited male or female CSWs with an age range of 18–60 at 3 districts of Surabaya from October to December 2012, and 3 male and 197 female CSWs agreed to be involved in the study (Table 1). Thirteen CSWs in urban area 1 worked at an exclusive night club, whereas 187 CSWs in urban area 2 or the rural area worked at inexpensive karaoke bars or brothels. The study participants were interviewed in Indonesian using a questionnaire that collected information on socio-demographic characteristics, sexual behavior, the general knowledge of sexually transmitted diseases (STDs), condom use in their previous commercial sex works and previous drug use. Ten milliliters of ethylenediaminetetraacetic acid (EDTA) anti-coagulated peripheral blood was collected from each participant. Plasma was then isolated from peripheral blood samples by centrifugation for 10 min at 2,000 rpm. In addition, peripheral blood mononuclear cells (PBMC) were isolated by density gradient centrifugation using Histopaque 1077 (Sigma-Aldrich, St. Louis, MO, USA). RNA and DNA were extracted from plasma and PBMC using the QIAamp Viral RNA Mini kit (Qiagen, Hilden, Germany) and GenElute Mammalian Genomic DNA Miniprep kit (Sigma-Aldrich), respectively.

**Table 1:** Demographic information of HIV-positive and -negative CSWs enrolled in this study.

	HIV-positive *				HIV-negative *			
	Total (%)	Urban area 1 (%)	Urban area 2 (%)	Rural area (%)	Total (%)	Urban area 1 (%)	Urban area 2 (%)	Rural area (%)
Sample number	22	0	12	10	178	13	112	53
Gender								
Male	0 (0.0) **	0 (0.0)	0 (0.0)	0 (0.0)	3 (1.7)	3 (23.1)	0 (0.0)	0 (0.0)
Female	22 (100.0)	0 (0.0)	12 (100.0)	10 (100.0)	175 (98.3)	10 (76.9)	112 (100.0)	53 (100.0)
Age (years old)								
<20	0 (0.0)	0 (0.0)	0 (0.0)	0 (0.0)	6 (3.4)	1 (7.7)	3 (2.7)	2 (3.8)
20–29	13 (59.1)	0 (0.0)	6 (50.0)	7 (70.0)	55 (30.9)	4 (30.8)	27 (24.1)	24 (45.3)
30–39	4 (18.2)	0 (0.0)	2 (16.7)	2 (20.0)	78 (43.8)	5 (38.5)	57 (50.9)	16 (30.2)
>40	4 (18.2)	0 (0.0)	4 (33.3)	0 (0.0)	32 (18.0)	3 (23.1)	24 (21.4)	5 (9.4)
No answer	1 (4.5)	0 (0.0)	0 (0.0)	1 (10.0)	7 (3.9)	0 (0.0)	1 (0.9)	6 (11.3)
Latest educational background								
Elementary school	10 (45.5)	0 (0.0)	8 (66.7)	2 (20.0)	78 (43.8)	0 (0.0)	63 (56.3)	15 (28.3)
Junior high school	7 (31.8)	0 (0.0)	2 (16.7)	5 (50.0)	47 (26.4)	2 (15.4)	25 (22.3)	20 (37.7)
High school	1 (4.5)	0 (0.0)	0 (0.0)	1 (10.0)	30 (16.9)	9 (69.2)	13 (11.6)	8 (15.1)
University	0 (0.0)	0 (0.0)	0 (0.0)	0 (0.0)	3 (1.7)	2 (15.4)	1 (0.9)	0 (0.0)
No answer	4 (18.2)	0 (0.0)	2 (16.7)	2 (20.0)	20 (11.2)	0 (0.0)	10 (8.9)	10 (18.9)
Duration of commercial sex work								
<3 months	1 (4.5)	0 (0.0)	0 (0.0)	1 (10.0)	20 (11.2)	0 (0.0)	11 (9.8)	9 (17.0)
3–12 months	6 (27.3)	0 (0.0)	2 (16.7)	4 (40.0)	31 (17.4)	0 (0.0)	17 (15.2)	14 (26.4)
1–3 years	12 (54.5)	0 (0.0)	7 (58.3)	5 (50.0)	60 (33.7)	1 (7.7)	43 (38.4)	16 (30.2)
>3 years	3 (13.6)	0 (0.0)	3 (25.0)	0 (0.0)	55 (30.9)	12 (92.3)	36 (32.1)	7 (13.2)
No answer	0 (0.0)	0 (0.0)	0 (0.0)	0 (0.0)	12 (6.7)	0 (0.0)	5 (4.5)	7 (13.2)
Number of clients per week								
1–3	7 (31.8)	0 (0.0)	3 (25.0)	4 (40.0)	32 (18.0)	7 (53.8)	20 (17.9)	5 (9.4)
4–6	6 (27.3)	0 (0.0)	6 (50.0)	0 (0.0)	60 (33.7)	3 (23.1)	48 (42.9)	9 (17.0)
>7	7 (31.8)	0 (0.0)	3 (25.0)	4 (40.0)	50 (28.1)	0 (0.0)	30 (26.8)	20 (37.7)
no answer	2 (9.1)	0 (0.0)	0 (0.0)	2 (20.0)	36 (20.2)	3 (23.1)	14 (12.5)	19 (35.8)
Awareness of sexually transmitted diseases								
Yes	0 (0.0)	0 (0.0)	0 (0.0)	0 (0.0)	10 (5.6)	10 (76.9)	0 (0.0)	0 (0.0)
No	19 (86.4)	0 (0.0)	12 (100.0)	7 (70.0)	144 (80.9)	0 (0.0)	109 (97.3)	35 (66.0)
No answer	3 (13.6)	0 (0.0)	0 (0.0)	3 (30.0)	24 (13.5)	3 (23.1)	3 (2.7)	18 (34.0)
Condom use								
Yes	3 (13.6)	0 (0.0)	0 (0.0)	3 (30.0)	10 (5.6)	0 (0.0)	0 (0.0)	10 (18.9)
No	19 (86.4)	0 (0.0)	12 (100.0)	7 (70.0)	160 (89.9)	13 (100.0)	111 (99.1)	36 (67.9)
No answer	0 (0.0)	0 (0.0)	0 (0.0)	0 (0.0)	8 (4.5)	0 (0.0)	1 (0.9)	7 (13.2)
Drug use								
Yes	0 (0.0)	0 (0.0)	0 (0.0)	0 (0.0)	14 (7.9)	11 (84.6)	3 (2.7)	0 (0.0)
No	22 (100.0)	0 (0.0)	12 (100.0)	10 (100.0)	159 (89.3)	2 (15.4)	108 (96.4)	49 (92.5)
No answer	0 (0.0)	0 (0.0)	0 (0.0)	0 (0.0)	5 (2.8)	0 (0.0)	1 (0.9)	4 (7.5)

*Plasma sample was tested for anti-HIV antibodies using a rapid diagnostic kit, and was then tested using 2 additional diagnostic kits to confirm the diagnosis of HIV infection.

**The proportion (%) of number of individuals in a question item is shown in parentheses.

### Sero-epidemiology

Plasma samples were tested for anti-HIV antibodies using a commercially available rapid diagnostic kit [ABON HIV 1/2/O Triline Human Immunodeficiency Virus Rapid Test Devices; Abon Biopharm (Hangzhou) Co., Ltd., Hangzhou, China], followed by an enzyme-linked immunosorbent assay (ELISA) system (HIV ASE 1+2; General Biologicals, Hsin Chu, Taiwan) and an immunochromatographic assay system [Anti-HIV 1/2 Device and Strip Test MONO (provided by the Ministry of Health, Indonesia); PT Askara Medical, Kota Bekasi, Indonesia] to confirm the diagnosis of HIV infection. In addition, hepatitis B core antibody (anti-HBc Ab) and hepatitis B surface antigen (HBsAg) in plasma samples were detected using a passive hemagglutination assay system (Mycel anti-rHBc) and a reverse passive haemagglutination assay system (Mycel II HBsAg), respectively, provided by the Institute of Immunology (Tokyo, Japan). Anti-HCV antibodies (Anti-HCV Ab) were detected by a particle agglutination assay system (Ortho HCV Ab PA test II; Fujirebio, Tokyo, Japan) and an ELISA system (Hepaliza anti HCV; Indec Diagnostics, Jakarta, Indonesia). Anti-GBV-C antibodies (Anti-GBV-C Ab) were detected using an ELISA system (HEPATITIS G- HGV/GBV-C; XpressBio, Thurmont, MD, USA).

### Amplification of HIV type 1 (HIV-1) genomic fragment

Viral RNA was reverse transcribed to cDNA using the SuperScript III First-Stand Synthesis kit (Invitrogen, Carlsbad, CA, USA) with the reverse primer, K-env-R1, 5′-CCAATCAGGGAAGAAGCCTTG-3′ [corresponding to nucleotide (nt) 9168 to 9148 of a HIV-1 reference strain, HXB2 (GenBank accession no. K03455)] [8]. The 288-base pair (bp) fragment of HIV-1 *pol* gene encoding a partial fragment of integrase and the 547-bp fragment of HIV-1 *env* gene encoding the C2-V3 regions of Env gp120 were then amplified by nested PCR using Ex Taq (Takara Bio, Shiga, Japan) and primer sets, as follows. For the amplification of viral *pol* gene fragment, UNIPOL5; 5′-TGGGTACCAGCACACAAAGGAATAGGAGGAAA-3′ (nt 4152 to 4183) and UNIPOL6; 5′-CCACAGCTGATCTCTGCCTTCTCTGTAATAGACC-3′ (nt 4934 to 4901) were used for the first PCR, and UNIPOL1; 5′-AGTGGATTCATAGAAGCAGAAGT-3′ (nt 4470 to 4492) and UNIPOL2; 5′-CCCCTATTCCTCCCCTTCTTTTAAAA-3′ (nt 4806 to 4781) were used for nested PCR [9], [10]. In addition, for amplification of the viral *env* gene, M5; 5′-CCAATTCCCATACATTATTGTGCCCCAGCTGG-3′ (nt 6858 to 6889) and M10; 5′-CCAATTGTCCCTCATATCTCCTCCTCCAGG-3′ (nt 7661 to 7632) were used for the first PCR, and M3; 5′-GTCAGCACAGTACAATGIACACATGG-3′ (nt 6948 to 6973) and M8; 5′-TCCTTGGATGGGAGGGGCATACATTGC-3′ (nt 7547 to 7521) were used for nested PCR [9]. The PCR conditions were as follows. For the 1st PCR of *pol* gene amplification, one cycle of 5 min at 94°C for denaturation; 35 cycles of 1 min at 94°C for denaturation, 1 min at 45°C for annealing and 1 min at 72°C for extension; and a final extension cycle of 5 min at 72°C were carried out. For the nested PCR of *pol* gene amplification, and the 1st and nested PCR of *env* gene amplification, the annealing temperatures were changed to 50°C, 55°C and 60°C, respectively. If a viral gene fragment failed to be amplified from the cDNA generated from viral RNA even after multiple attempts, it was amplified instead from DNA extracted from PBMC. In order to examine the genomic fragment of the major viral population in a sample, PCR products amplified at the end-point dilution of cDNA or DNA templates were subjected to sequencing analysis.

### Sequencing analysis and HIV-1 subtyping

Sequencing analysis of the amplified HIV-1 genomic fragment was carried out using the BigDye Terminator v1.1 Cycle Sequencing kit with an ABI PRISM310 genetic analyzer (Applied Biosystems, Foster City, CA, USA), and data were assembled using Genetyx ver 10 software (Genetyx, Tokyo, Japan). HIV-1 subtyping was carried out using the Recombinant Identification Program (RIP) available at the website of the HIV sequence database (http://www.hiv.lanl.gov/). In addition, phylogenetic analysis of HIV-1 *pol* and *env* gene fragments was conducted using MEGA5.2 software [11], after multiple alignment using the Clustal W algorithm and manual editing. The nucleotide distance matrices generated using the Kimura two-parameter model [12] were used to construct a phylogenetic tree by the neighbor-joining method [13]. All gaps and missing data were stripped before computing the distance matrices. Bootstrap values (1,000 replicates) [14] for relevant nodes were reported on a representative tree.

### Statistical analysis

Statistical analysis was performed using Fisher's exact test for categorical variables. Briefly, a 2×2 contingency table on the selected data was constructed, and the 2-tailed p-value was calculated using QuickCalcs (GraphPad software; http://www.graphpad.com/quickcalcs/). P values less than or equal to 0.05 were considered to be significant.

### Nucleotide sequence accession numbers

The nucleotide sequences of the viral gene fragments have been deposited in the GenBank database under accession numbers KF147334-KF147375.

## Results

### High prevalence of HIV infection among CSWs residing in Surabaya

We collected 200 peripheral blood samples from CSWs residing in 3 districts of Surabaya. Sero-epidemiological tests revealed that 22 out of 200 CSWs (11%) were HIV positive (Table 1). The HIV prevalence rates of CSWs in urban area 1, urban area 2 and the rural area were 0%, 9.7% and 15.9%, respectively, showing the regional difference in the prevalence rate of HIV infection. Demographic information for HIV-positive and -negative CSWs is shown in Table 1. According to the questionnaire for study participants, more than half of HIV-positive CSWs were young (<30 years old) women who had been involved in commercial sex work for 1–3 years (Table 1). In addition, none of the HIV-positive CSWs were IDUs, suggesting the sexual transmission of HIV in commercial sex work. Most CSWs involved in this study had a low educational background and showed low awareness of STDs, including HIV infection (Table 1). Thirteen CSWs residing in urban area 1 had a relatively higher educational background as well as showing higher awareness of STDs than the remaining 187 CSWs residing in urban area 2 or in the rural area (Table 1). However, most CSWs (86.4%) residing in the 3 districts had unprotected sex (without condom use) with their clients (Table 1). In addition, no statistically significant difference was observed in the percentage of condom use between HIV-positive and –negative CSWs.

### HIV-1 subtyping

**Figure 1:**
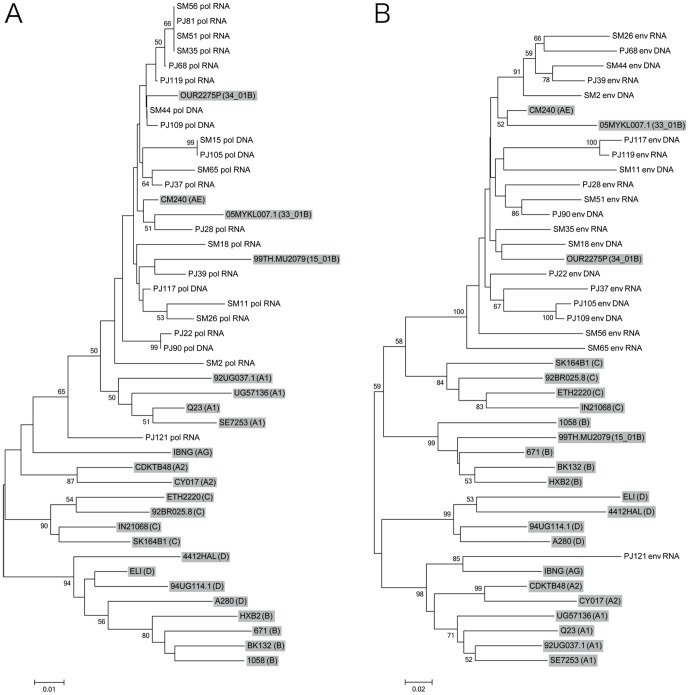
Phylogenetic relationship of HIV-1 *pol* and *env* gene sequences. Phylogenetic trees were generated for newly sequenced HIV-1 *pol* (A) and *env* (B) genes together with the corresponding viral gene of reference HIV-1 strains representing subtype A1 (A1), subtype A2 (A2), subtype B (B), subtype C (C), subtype D (D), CRF01_AE (AE), CRF02_AG (AG), CRF15_01B (15_01B), CRF33_01B (33_01B) and CRF34_01B (34_01B) (shown with a gray background). Bootstrap values are shown when the values are >50. Scale bar represents 0.01 (A) or 0.02 nucleotide substitutions per site (B). The nucleotide sequences of *pol* and *env* genes determined in this study have been deposited in the GenBank database under accession numbers KF147334-KF147375.

The partial fragments of HIV-1 *pol* and *env* genes were PCR- or RT-PCR-amplified and subjected to sequencing analysis. Viral subtyping using the RIP program revealed that most amplified viral *pol* and *env* gene fragments were classified into CRF01_AE viral genes, except those derived from sample PJ121 (Table 2). HIV-1 *pol* and *env* gene fragments derived from PJ121 were classified into CRF01_AE and subtype A1, respectively, by RIP (Table 2), while these viral genes were located near the reference strains of subtype A1 (*pol* gene, Fig. 1A) and CRF02_AG (*env* gene, Fig. 1B) on phylogenetic trees. These results suggest that most CSWs were infected with CRF01_AE viruses, whereas PJ121 was infected with an unique recombinant form of HIV-1, subtype A1 or CRF02_AG virus.

**Table 2:** Viral subtype and CRF detected in the blood samples of HIV-1-positive CSWs.*

	HIV-1 gene	
sample ID	*pol*	*env*
SM2	CRF01_AE	CRF01_AE
SM11	CRF01_AE	CRF01_AE
SM15	CRF01_AE	nd **
SM18	CRF01_AE	CRF01_AE
SM26	CRF01_AE	CRF01_AE
SM35	CRF01_AE	CRF01_AE
SM44	CRF01_AE	CRF01_AE
SM51	CRF01_AE	CRF01_AE
SM56	CRF01_AE	CRF01_AE
SM65	CRF01_AE	CRF01_AE
PJ22	CRF01_AE	CRF01_AE
PJ28	CRF01_AE	CRF01_AE
PJ37	CRF01_AE	CRF01_AE
PJ39	CRF01_AE	CRF01_AE
PJ68	CRF01_AE	CRF01_AE
PJ81	CRF01_AE	nd
PJ90	CRF01_AE	CRF01_AE
PJ105	CRF01_AE	CRF01_AE
PJ109	CRF01_AE	CRF01_AE
PJ117	CRF01_AE	CRF01_AE
PJ119	CRF01_AE	CRF01_AE
PJ121	CRF01_AE	Subtype A1

*HIV-1 *pol* and *env* genes were amplified and subjected to sequencing analysis.

Viral subtyping was carried out using the Recombinant Identification Program (RIP).

**HIV-1 *env* gene failed to be amplified; therefore, viral subtyping was not carried out.

### Prevalence of HBV, HCV and GBV-C infection among CSWs in Surabaya

Sero-epidemiological tests for HBV infection revealed that 8 (4%) and 128 (64%) out of 200 CSWs were seropositive for HBsAg and anti-HBc Ab, respectively (Table 3). In addition, 8 (4.5%) out of 178 HIV-negative CSWs, consisting of 5 (4.5%) and 3 (5.7%) individuals in urban area 2 and the rural area, respectively, were seropositive for HBsAg, while no individual was HBsAg-seropositive among 22 HIV-positive CSWs (Table 3), suggesting that no HIV/HBV co-infection was established among CSWs involved in this study, if HBsAg was considered to be a marker of HBV infection. In addition, 17 (77.2%) of 22 HIV-1-positive CSWs, consisting of 9 (75%) and 8 (80%) individuals in urban area 2 and the rural area, respectively, were seropositive for anti-HBc Ab, whereas 111 (62.4%) of 178 HIV-negative CSWs, consisting of 7 (53.8%), 68 (60.7%) and 36 (68%) individuals in urban area 1 and 2, and the rural area, respectively, were seropositive for anti-HBc Ab (Table 3). These results suggested that CSWs were at a high risk of exposure to HBV, although chronic HBV infection was less frequently established. No statistically significant differences were observed in anti-HBc Ab positivity among CSWs in the 3 districts as well as between HIV-positive and -negative CSWs (Table 3). In addition, among 200 CSWs, an individual was seropositive for anti-HCV Ab, whereas no individual was seropositive for anti-GBV-C Ab (Table 3), suggesting the low prevalence of HCV and GBV-C infection among CSWs involved in this study.

**Table 3:** Sero-prevalence of HBsAg, anti-HBc Ab, anti-HCV Ab and anti-GBV-C Ab among CSWs residing in Surabaya.*

	All participants				HIV-positive **				HIV-negative **			
	Total (n = 200)	Urban area 1 (n = 13)	Urban area 2 (n = 124)	Rural area (n = 63)	Total (n = 22)	Urban area 1 (n = 0)	Urban area 2 (n = 12)	Rural area (n = 10)	Total (n = 178)	Urban area 1 (n = 13)	Urban area 2 (n = 112)	Rural area (n = 53)
HBsAg -positive	8 (4.0) ***	0 (0.0)	5 (4.0)	3 (4.8)	0 (0.0)	0 (0.0)	0 (0.0)	0 (0.0)	8 (4.5)	0 (0.0)	5 (4.5)	3 (5.7)
Anti-HBc Ab-positive	128 (64)	7 (53.8)	77 (62.1)	44 (70.0)	17 (77.2)	0 (0.0)	9 (75.0)	8 (80.0)	111 (62.4)	7 (53.8)	68 (60.7)	36 (68.0)
Anti-HCV Ab-positive	1 (0.5)	0 (0.0)	1 (0.8)	0 (0.0)	0 (0.0)	0 (0.0)	0 (0.0)	0 (0.0)	1 (0.6)	0 (0.0)	1 (0.9)	0 (0.0)
Anti-GBV-C Ab-positive	0 (0.0)	0 (0.0)	0 (0.0)	0 (0.0)	0 (0.0)	0 (0.0)	0 (0.0)	0 (0.0)	0 (0.0)	0 (0.0)	0 (0.0)	0 (0.0)

*Plasma sample was tested for HBsAg, anti-HBc Ab, anti-HCV Ab and anti-GBV-C Ab using commertially available diagnostic kits.

**Plasma sample was tested for anti-HIV antibodies using a rapid diagnostic kit, and was then tested using 2 additional diagnostic kits to confirm the diagnosis of HIV infection.

***The sero-prevalence rate (%) among a group of CSWs indicated is shown in parentheses.

## Discussion

Our study revealed that the prevalence rate of HIV infection among CSWs residing in Surabaya was significantly higher (11%) than that among the general population in Indonesia (0.2–0.4%) [1]. In addition, most (>86.4%) HIV-infected CSWs showed low awareness of STDs, including HIV infection, and 86.4% of them had unprotected sex with their clients previously. We failed to collect more than 13 samples from CSWs working at an exclusive night club in urban area 1 of Surabaya. Therefore, it may be difficult to conclude statistically; however, our results suggest a regional difference in the HIV prevalence rate among CSWs in Surabaya. HIV prevalence was high among female CSWs working at inexpensive karaoke bars or brothels in urban area 2 and the rural area, which are part of the largest prostitution complex in Southeast Asia. A previous study conducted in Jakarta and Bali in 2006–2008 revealed that the HIV prevalence rate among female CSWs residing in these Indonesian cities was 7.7% [15]. In addition, a previous report show that the HIV prevalence rate in female CSWs in several Indonesian cities was 8.2–10.5% [16], [17], while it was 6.3% in Surabaya in 2007 [17]. Our and previous results suggested that the prevalence of HIV infection among female CSWs in Surabaya is stable or has inclined recently. Studies conducted in Phnom Penh and Hai Phong in Vietnam in 2007 revealed that the HIV prevalence rate among female CSWs in Vietnam was 23.1% [18], [19]. In addition, studies conducted in Bangkok, Thailand in 2004–2007 revealed that the HIV prevalence rates of venue-based female CSWs and non-venue-based female CSWs were 4.2–12.5% and 22.8–45.8%, respectively [20], [21]. These reports show that the prevalence of HIV infection is markedly high among female CSWs in many Southeast Asian countries. Considering the currently growing epidemic of HIV infection in Indonesia, it is necessary to accumulate more epidemiological data on HIV infection among CSWs in major cities all over Indonesia.

HIV-1 is characterized by extensive genetic heterogeneity and is divided into four groups: M (major), O (outlying), N (new or non-M, non-O) and P (pending). The viruses in group M, which are responsible for the worldwide HIV pandemic, are further classified into many subtypes and circulating recombinant forms (CRFs) [22]. While subtype B of HIV-1 is the predominant subtype in the Americas, Europe and Australia, there is a growing epidemic of non-B subtypes and CRFs in Africa and Asia. Recently, new CRFs, CRF33_01B and CRF34_01B, were isolated in Indonesia [23], [24]; therefore, in order to survey the possible appearance of previously undetected types of HIV-1, we performed viral subtyping in this study. We detected the genomic fragments of CRF01_AE viruses, a predominant CRF in Southeast Asia [22], in most samples derived from HIV-positive CSWs (Table 2), suggesting that CRF01_AE viruses are still the predominant strain of HIV-1 in Surabaya, Indonesia. However, viral *pol* and *env* gene fragments derived from a study participant, PJ121, were classified into CRF01_AE and subtype A1, respectively, by RIP (Table 2), while these viral genes were located near the reference strains of subtype A1 (*pol* gene, Fig. 1A) and CRF02_AG (*env* gene, Fig. 1B) on phylogenetic trees, suggesting the emergence of a unique recombinant form of HIV-1, subtype A1 or CRF02_AG in Surabaya. We consider that is necessary to reveal the genotype of this virus in detail in a future study.

A previous study revealed that the prevalence rate of HBsAg was 6.7% among the general population in Surabaya [25]. In addition, the prevalence rate of anti-HBc Ab among HBsAg-negative individuals was 43.4% in Java and the Sumatra Islands in Indonesia [26]. Our results showed that the prevalence rate of anti-HBc Ab among CSWs were higher (64%) than that in a previous study [26], although the prevalence rate of HBsAg among CSWs (4%) was comparable to or even lower than that among the general population in Surabaya [25]. We consider that such a high prevalence of anti-HBc Ab might be due to the low awareness of STDs and the high-risk behavior of CSWs for HBV infection, such as an unprotected sex with clients. In addition, previous reports showed that the prevalence rates of HBsAg and anti-HBc Ab were 3.2–15.3% and 30.2% among HIV-infected individuals in Indonesia, respectively [27], [28]. Our results revealed no HIV/HBV co-infection (anti-HIV Ab and HBsAg double positive), in spite of the high prevalence of anti-HBc Ab among CSWs (Table 3). However, a previous report showed a high prevalence (24–31%) of occult HBV infection (with a low level of HBV DNA without a detectable HBsAg) among HIV-infected, anti-HBc Ab-seropositive individuals [29], [30]. In addition, genomic DNA of HBV was detected in 32 out of 100 (32%) HIV-infected, HBsAg-seronegative individuals in Surabaya [27]. Based on these previous results, some HIV-positive, anti-HBc Ab-seropositive, and HBsAg-seronegative CSWs potentially carry HBV as an occult infection. Therefore, we consider that there is a need for a follow-up study of the HIV-infected, anti-HBc Ab-seropositive CSWs involved in this study.

Previous studies revealed that the prevalence rates of HCV and GBV-C infection among the general population in Surabaya were 2.3% and 2.7%, respectively [31], [32]. In addition, the prevalence rates of HCV and GBV-C infection were 34.1% and 88.8% among HIV-infected individuals in Yogyakarta, respectively [28], [33], whereas that of HCV infection was 46.6% among HIV-infected individuals in Surabaya, Indonesia (Utsumi et al., unpublished data). In contrast to these reports, we observed a low prevalence of HCV and GBV-C infection among CSWs in Surabaya (Table 3). This discrepancy may be because most CSWs involved in this study were not IDUs (Table 1), whereas 44.4–62.7% of HIV-infected individuals involved in previous studies were IDUs [27], [28]. Our and previous results were consistent with reports that HCV and GBV-C mainly transmit through the sharing of needles and syringes, rather than through sexual contact [33], [34], [35], [36].

Finally, our study revealed a high prevalence of HIV-1 infection and the low awareness of STDs among CSWs (Table 1). In addition, most CSWs had unprotected sex with their clients (Table 1); therefore, the clients have a potential role in spreading HIV to the general population of Indonesia. Therefore, we consider the necessity of surveillance studies of HIV infection, not only among high-risk groups such as CSWs, but also among low-risk groups for HIV infection such as pregnant women. In addition, considering the rapidly growing epidemic of HIV infection in Indonesia, it is necessary to conduct a follow-up surveillance study of CSWs. We hope to be involved in these future studies and to provide more epidemiological data on HIV infection that may be essential for the development and implementation of efficient disease control and prevention programs in Indonesia.
